# Quantized Inverse Design for Photonic Integrated Circuits

**DOI:** 10.1021/acsomega.4c10958

**Published:** 2025-01-27

**Authors:** Frederik Schubert, Yannik Mahlau, Konrad Bethmann, Fabian Hartmann, Reinhard Caspary, Marco Munderloh, Jörn Ostermann, Bodo Rosenhahn

**Affiliations:** † Institute for Information Processing, Leibniz University, 30167 Hannover, Germany; ‡ PhoenixD, Leibniz University, 30167 Hannover, Germany

## Abstract

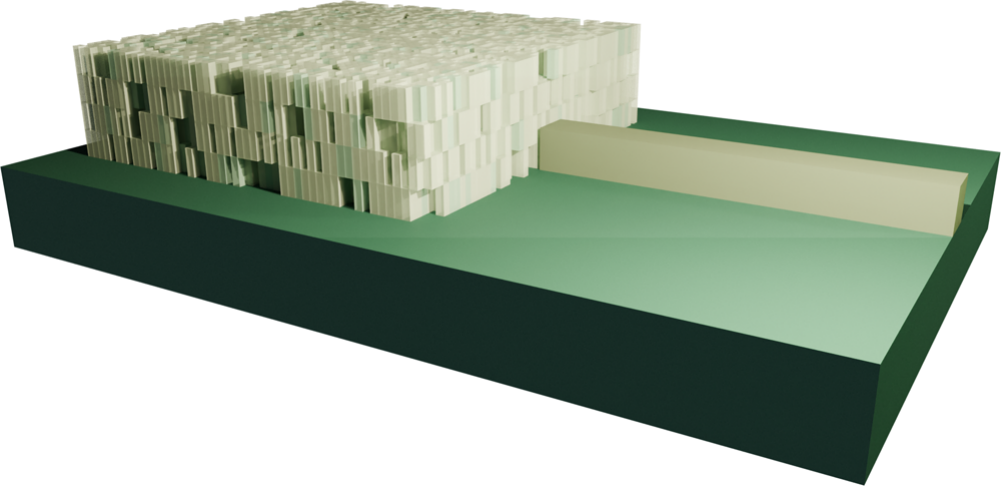

The inverse design of photonic integrated circuits (PICs) presents distinctive computational challenges, including their large memory requirements. Advancements in the two-photon polymerization (2PP) fabrication process introduce additional complexity, necessitating the development of more flexible optimization algorithms to enable the creation of multimaterial 3D structures with unique properties. This paper presents a memory efficient reverse-mode automatic differentiation framework for finite-difference time-domain (FDTD) simulations that can handle complex constraints arising from novel fabrication methods. Our method is based on straight-through gradient estimation that enables nondifferentiable shape parametrizations. We demonstrate the effectiveness of our approach by creating increasingly complex structures to solve the coupling problems in PICs. The results highlight the potential of our method for future PIC design and practical applications.

## Introduction

The availability of commercial two-photon polymerization (2PP) printers^1,2^ has led to an increased interest in the production and optimization of photonic integrated circuits (PICs) based on polymers. In comparison to silicon-based PICs, polymer-based PICs offer advantages in terms of prototyping and material costs.^3^ However, there are also disadvantages, such as the high attenuation of the polymers compared to silicon-based components.^4^ A primary constraint is their small refractive index contrast, which necessitates the use of relatively large devices in comparison to the wavelength.^5^ Furthermore, light is coupled in and out using butt coupling or gratings^6,7^ which have a large spatial footprint and further complicate the optical circuits. This limitation affects the complexity of the inverse design process using techniques such as the finite-differences time-domain (FDTD) method^8^ since the interaction of light and material must be modeled at sufficiently high resolution over the simulated volume. Developments such as the multimaterial 2PP process can alleviate this problem of low refractive contrast by enabling more complex ways of manipulating the incoming light.^9,10^ Moreover, it also allows for the integration and optimization of active components due to the possibility of incorporating quantum dots into the polymer matrix.^11^ Nevertheless, the inverse design of such structures presents a challenge. Other proposed methods, such as the adjoint method,^12,13^ are not directly applicable as they require the analytical derivation of operators for more complicated design spaces. The advancements in highly parallel hardware such as GPUs and the respective efficient software^14−16^ have made reverse-mode automatic differentiation (AD) a viable option, despite its limitations in terms of memory requirements when using a naive implementation. This paper proposes a memory efficient framework that combines the flexibility and scalability of automatic differentiation with the generality of the FDTD method. Our method employs an efficient implementation of reverse-mode AD using the time-reversibility of Maxwell’s equations. The method scales from a single machine to whole GPU clusters and is able to optimize a wide range of objective functions, design parameter spaces, and constraints. The flexibility of our method results from the straight-through gradient estimator which has been successfully applied to the 2D optimization problem of silicon PICs.^17^ We demonstrate the versatility of our method by optimizing the design of several polymer-based vertical couplers, which are essential for the implementation and fabrication of PICs.

In summary, our main **contribution** is a memory efficient implementation of reversed-mode automatic differentiation. This enables the creation of a general quantization-based framework to integrate arbitrary constraints in 3D into the inverse design process of large-scale PICs. The framework scales from single GPUs to GPU clusters and is published as open source to spur further research in this area.^1^ We demonstrate the capabilities of our approach by the inverse design of several coupling devices that range from a simple silicon coupler to a multimaterial polymer coupler with complex fabrication constraints. Our results indicate the potential of multimaterial 2PP for the fabrication of PICs.

## Methods

Our work is based on the finite-differences time-domain method, which we briefly introduce in the following section. We also provide an overview of the inverse design problem for PICs, including a discussion of the capabilities and limitations of previous approaches that use the adjoint method or automatic differentiation. Finally, we introduce our method to integrate arbitrary constraints into the inverse design process.

### Finite-Differences Time-Domain Method

Our framework is based on the finite-differences time-domain (FDTD)^8^ method. FDTD solves the classical electrodynamics description of Maxwell’s equations^18^ using leapfrog integration. The electric and magnetic fields **E** and **H** are discretized and arranged in a staggered Yee Lattice. Given an initial state, the fields are integrated in discrete time steps in an interleaved pattern. The time step duration Δ*t* is determined according to the Courant Levy stability conditions,^19^ which ensure that information does not propagate faster than the speed of light. The updated equations for the fields are given by Maxwell’s equations:
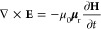
1
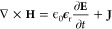
2where **E** and **H** are discretized in space and time. The fields in the simulation are modulated by permittivity and permeability tensors, **ϵ**_r_ and **μ**_r_, which are also discretized onto the Yee grid. When the fields are excited by an electric source **J**, the energy follows the dynamics of Maxwell’ equations and travels in waves throughout the simulation. To simulate an infinitely large space, we apply convolutional Perfectly Matched Layers (CPMLs) at the boundaries of our simulation.^20^ Accurately modeling the fields for a given source wavelength λ_0_ sets bounds on the spatial resolution Δ***s*** that should be approximately λ_0_/10^21^ but has to be adapted to the specific problem. For metrics that combine the electric and magnetic fields, such as the Poynting flux **P** = **E** × **H**, the fields from the leapfrog integration have to be synchronized in time and interpolated on the same spatial grid coordinates from the Yee lattice.

### Inverse Design for PICs

The inverse design of PICs can generally be formulated as a constrained *topology optimization* problem. In the context of polymer-based PICs, the design space is the discrete distribution of polymer in a fixed volume to maximize a given objective function . Many methods are gradient-based, i.e., they use the local sensitivity of the objective with respect to the design parameters θ to guide their optimization. A common design parametrization in photonic inverse design is the permittivity tensor of the design region from which the manufacturable device can be derived. The predominant method for gradient-based optimization in the context of FDTD simulations is the adjoint variable method^12,13,22^ due to its efficiency and scalability. This, however, restricts the design space to smooth and differentiable functions such as level-set methods^23,24^ where the gradients of the adjoint fields with respect to the parameters can be derived analytically.^25−30^ The recently proposed time-reversal direct differentiation method^31^ suffers from the same limitations. A method to circumvent this constraint is the combination of the adjoint method with automatic differentiation.^32,33^ This releases the user from deriving the analytical gradients and allows for a more flexible design space. However, all of the above methods are limited to continuous design spaces. Some works use discrete optimization methods such as Ballew et al.^34^ but introduce additional complexity via an additional inner optimization problem and are not as efficient as first-order gradient-based methods.

In this work, we propose a framework to efficiently design devices with arbitrary constraints. Following Schubert et al.^17^ and Paolini et al.,^35^ we apply the straight-through gradient estimator (STE)^36,37^ to implement our constrained optimization problem
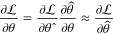
3The STE allows us to backpropagate gradients through nondifferentiable functions *q* by replacing the transformation of the gradient in the backward pass with the identity function. This introduces a gradient error but leads to approximately equivalent latent parameter movement when using adaptive learning rate optimizers^38^ like Adam.^39^ The STE method, however, has limitations as it might only provide a first-order approximation of the true gradient.^40^ When the quantization function leads to complex field variations around the design region, resulting in significantly altered gradient directions, the optimization process may stagnate. This limitation becomes particularly pronounced when dealing with quantization functions that involve long-range dependencies, such as connectivity constraints with an outer volume.

**Figure 1:**
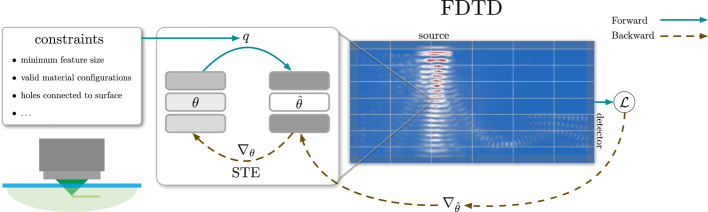
Overview of the proposed method. The fabrication constraints are encoded in the quantization mapping *q* which transforms the latent parameters θ into discrete realization θ̂. The fields are propagated via the FDTD simulation and the gradients of the objective function  are backpropagated through the simulation. Finally, the gradients are transformed via the straight-through estimator (STE) to update the latent parameters.

The STE is particularly useful for the FDTD simulation, as it allows us to backpropagate gradients through the discrete design parameters θ̂ = *q*(θ) of the simulation into the continuous latent parameters θ. In our framework, we choose the STE instead of a continuous latent variable with subsequent binarization due to our focus on polymer-based multimaterial devices with complex constraints such as the connectivity of gaps inside the device with the outer volume of air to remove the unpolymerized photoresist. An overview of our framework is shown in Figure 1.
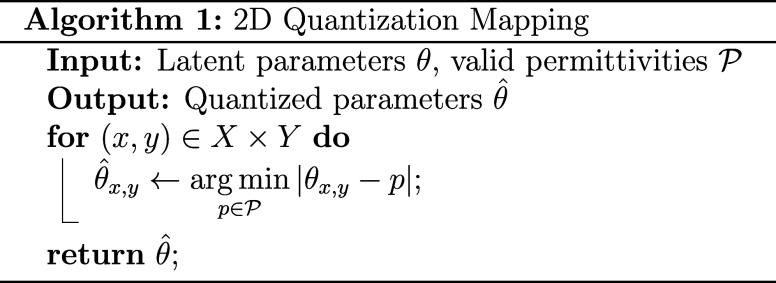


In this work, we use a single scalar  as a latent variable for each voxel in the topology optimization region. Using the quantization function *q*, latent variables θ are mapped to quantized parameters θ̂. Specifically, the condition  has to hold for all (*x*, *y*, *z*) ∈ *X* × *Y* × *Z*, where  is the set of valid permittivities. For example, when designing single material PIC using the ma N 1400 Series polymer,^41^ valid permittivities would be  representing the binary choice between air or material. The quantization mapping *q* to the closest valid permittivity is displayed in Algorithm 1. The more complex quantization constraints for 2.5D and 3D are described in later sections.
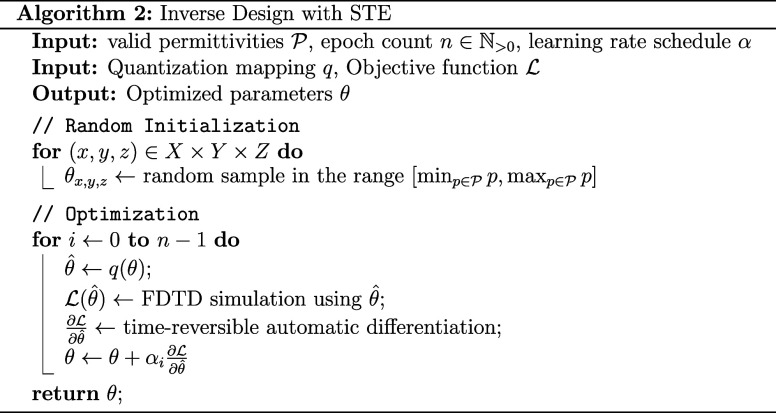


In Algorithm 2, our full optimization algorithm is shown. First, the latent parameters θ are initialized randomly between the minimum and maximum valid permittivity. Then, an optimization loop performs a gradient ascent for *n* iterations. In each iteration, the parameters θ are quantized and run through the FDTD simulation. Using a memory efficient time-reversible implementation of automatic differentiation, we calculate the gradient  of the objective function  with respect to the quantized parameters. With the STE approximation, we can use this gradient to update the latent parameters. We determine the learning rates α_*i*_ using the Adam optimizer^39^ with Nesterov momentum^42^ and cosine-decay learning rate schedule with linear warmup.^43^

#### Memory Efficient Implementation

Calculating the gradient through an FDTD simulation using direct differentiation can be very memory intensive. A naive implementation requires the electric and magnetic fields to be saved after every time step. We circumvent this problem by using the time-reversible nature of Maxwell’s equations.^45^ With this technique, it is possible to calculate an inverse time step of the FDTD simulation that transforms the electric and magnetic fields at time step *t* + 1 back to the fields at time step *t*. As a result, only the fields of two-time steps need to be kept in memory at any time.^2^ Unfortunately, the Perfectly Matched Layers (PML) at the boundary are not time-reversible. Therefore, it is necessary to save the electric and magnetic fields at the boundary between the PML and simulation volume for every time step to prevent information loss. Even with this downside, the time-reversible gradient computation is more memory efficient than the naive implementation, because saving the 2D slices between PML and simulation volume requires much less memory than saving the full 3D simulation volume. Previously, this technique has been successfully demonstrated by Tang et al.,^31^ but has never been integrated into a powerful automatic differentiation framework like JAX.^16^ The flexibility of automatic differentiation and the memory efficiency of time-reversible gradient computation result in a user-friendly workflow on relatively small hardware.

#### Demonstration

**Figure 2:**
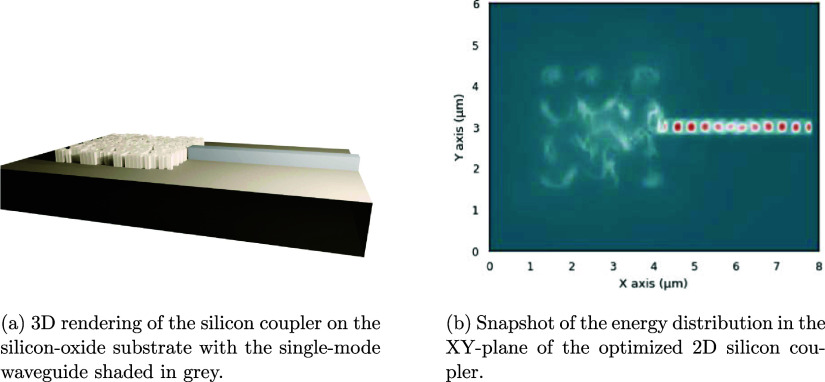
Reproduction of the silicon coupler from Shen et al.^44^ The structure consists of 30 × 30 cells of size 100 nm^2^ that are either silicon or air. The device is optimized for a wavelength of 1550 nm and a structure height of 300 nm.

To demonstrate our framework in the domain of silicon photonics, we reproduce the coupling device from Shen et al.^44^ The device consists of 30 × 30 square cells with a side length of 100 nm that are either silicon or air. The objective is the transfer efficiency from a free space planar wave into a single-mode silicon waveguide with a diameter of 400 nm. The device is optimized for a wavelength of 1550 nm and a structure height of 300 nm. By starting a new optimization from random parameters, we match the reported results by Shen et al.^44^ with a loss of −2.7 dB compared to their −3 dB. The resulting coupler is shown in Figure 2.

## Results and Discussion

To demonstrate the capabilities of our framework, we present three different examples of PICs. The goal is to find a solution to the coupling problem, i.e., the efficient transfer of light from an external source into the photonic circuit.

**Figure 3:**
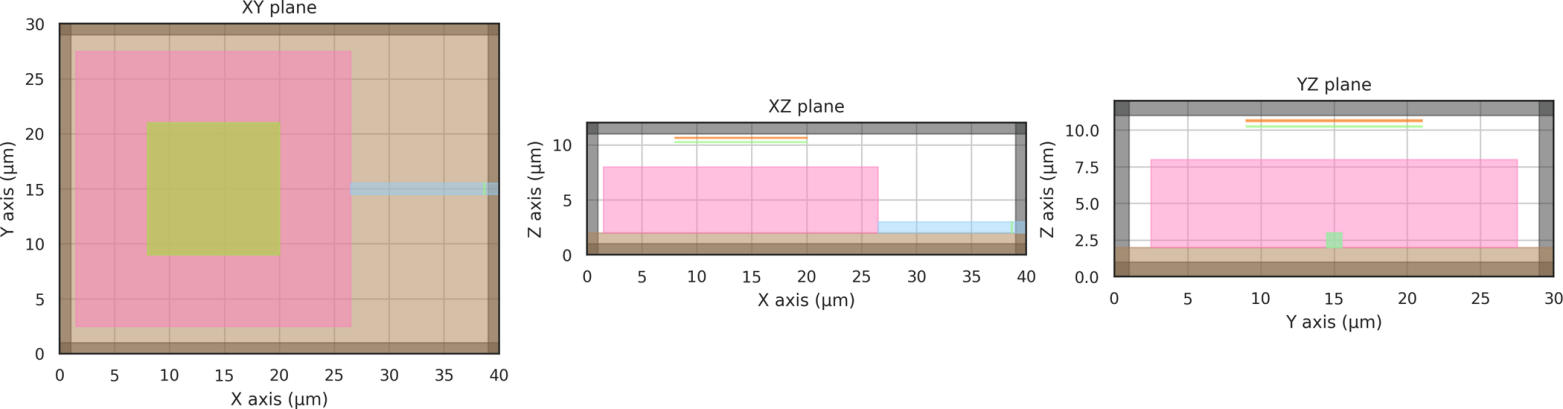
Setup of the vertical coupler with the free field source region (orange) above the device (pink). The device is connected to the output waveguide (blue) and is placed on top of a fused silica substrate (brown). Its efficiency is evaluated as the average Poynting flux at the end of the waveguide (green) normalized by the incident Poynting flux at the detector below the source. The reference plane on the input side (green) is placed at a small distance to the device to minimize the influence of reflections from the device during the optimization.

They range from a simple single-material vertical coupler with 2D and 2.5D structures to a full 3D two-polymer vertical coupler. We refer to 2.5D as a 2D-pixel grid, where each pixel may be extruded to a different height in the third dimension.^46−48^ The general setup of the coupler is shown in Figure 3. We simulate a continuous source with a Gaussian intensity distribution of 4 μm radius and a wavelength of 1.55 μm at a grid resolution of 100 nm. The source is placed at the region indicated in Figure 3 (orange rectangle in the *XY* plane) with random lateral offsets of 2 μm. Our setup consists of a 3D simulation volume of 40 μm × 30 μm × 12 μm, which results in 14.4 M grid cells. Note that the device is larger than the silicon coupler by a factor of about 10 due to the low refractive index contrast of the polymer. The device and waveguide are placed on a fused silica substrate with a refractive index of 1.45. The objective function is the average Poynting flux at the end of the output waveguide normalized by the incoming Poynting flux at the detector below the source. The output waveguide is rectangular, with a width and height of 1 μm.

The simulation is run for 250 fs with a Courant factor of 0.99, which results in 1311 FDTD iterations. We average the gradients over 3 simulations with random lateral source offsets to simulate calibration errors in our optimization process. Each optimization iteration took approximately 110 s on a single NVIDIA A100.

For these optimizations, the design parameters θ of the coupler are the distributions of the polymer in the volume of the device. The device is constrained by the minimum feature size in the *XY* plane of 500 nm and either the possibility of using the full 3D design space or just the 2D and 2.5D solutions that are common in the literature. For 2.5D structures, we impose a constraint that prevents any overhanging elements. While the 3D design space offers more flexibility, it can potentially generate impractical designs with floating materials or cavities that would trap unpolymerized material. To ensure feasibility in 3D designs, we incorporate these physical constraints directly into the quantization mapping.

### Single Material Vertical Coupler in 2D and 2.5D

**Figure 4:**
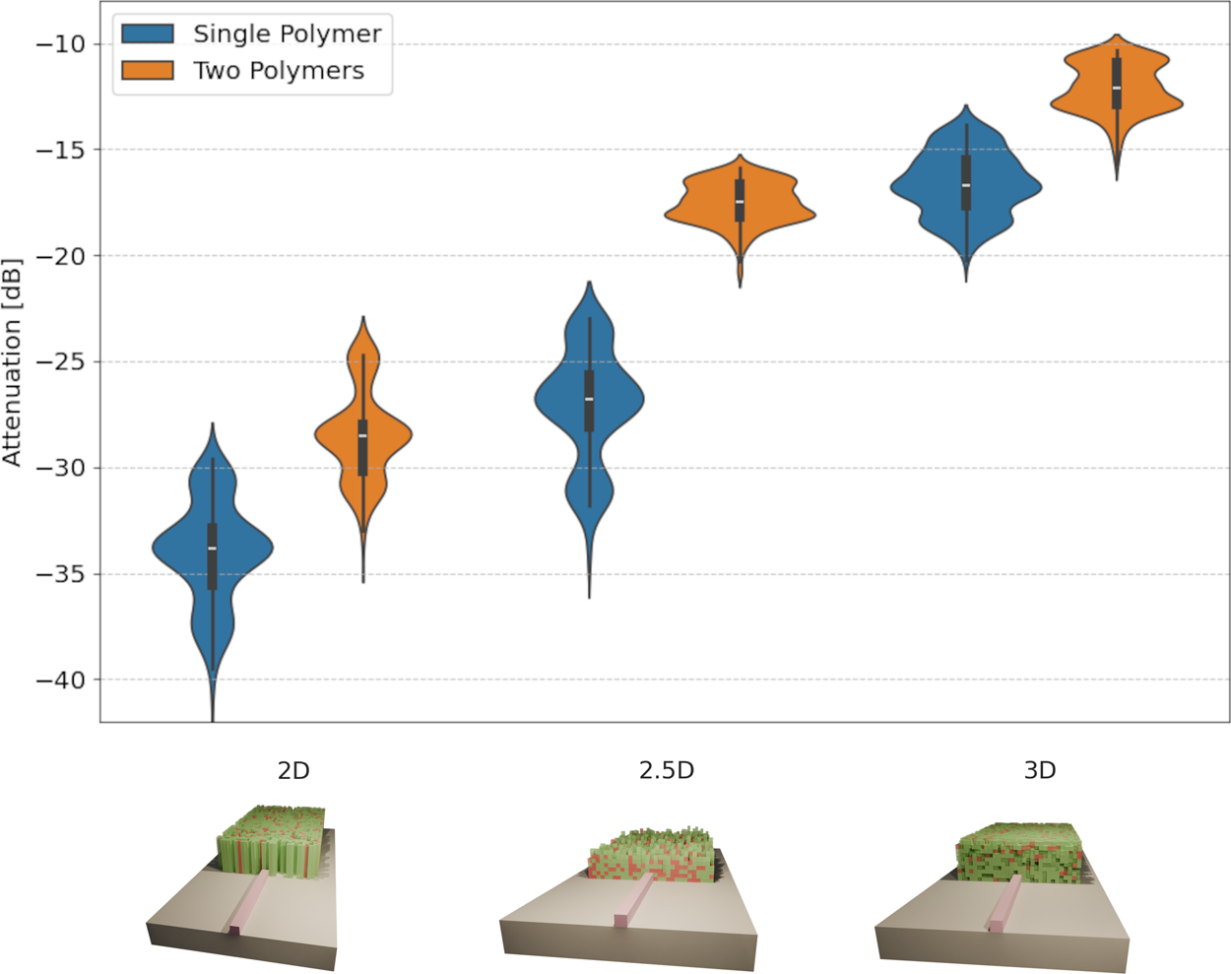
Comparison of coupler designs showing increasing efficiency benefits from a single polymer and two polymers for different design constraints. The median is computed over 50 evaluations per design and is due to the angle of the incoming light, as well as the transverse displacement of the light source. The cavities in the 3D structures are always connected to the outer volume, which allows for the removal of the unpolymerized photoresist.

**Figure 5:**
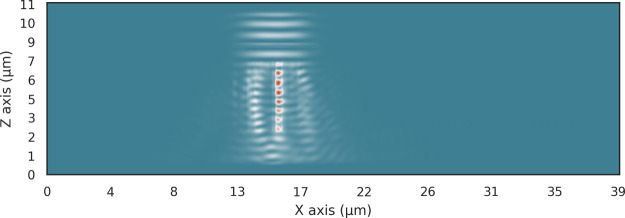
Energy distribution of the optimized 2D single material vertical coupler with a height of 5 μm and a final attenuation of −34 dB. Almost no light is coupled into the waveguide to the right due to the low refractive index contrast.

The efficiency of a vertical coupler depends on its ability to manipulate incoming light via changes in the refractive index. In this experiment, the number of ways for the coupler to interact with the light is limited by the single material design using the ma N 1400 Series polymer (Figure 4).^41^ The results in Figure 5 show that the 2D distribution of the polymer does not provide the required degrees of freedom to guide the light into the waveguide. With an attenuation of −34 dB, almost all the light is lost to the substrate below the device.

**Figure 6:**
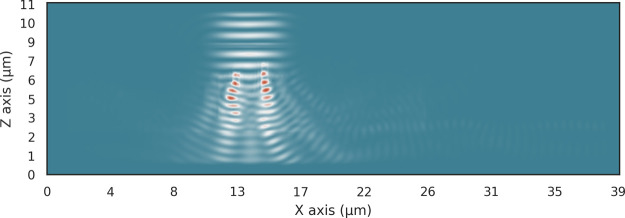
Energy distribution of the optimized 2.5D single material coupler. The Gaussian plane source above the device is visibly coupled into the single-mode waveguide to the right. However, the efficiency of −29 dB remains low, with significant energy loss to the substrate below and the surrounding volume.

To improve the efficiency and to demonstrate the versatility of our quantization method, we extend our design space to 2.5D. The results in Figure 6 demonstrate that the efficiency of the 2.5D single material coupler is still insufficient, with almost all of the energy being lost to the substrate and the volume around the device. Thus, this experiment highlights the need for more advanced designs with higher degrees of freedom to achieve efficiencies comparable to those in silicon PICs.

### Two-Polymer Vertical Coupler

In this experiment, we demonstrate the inverse design of multiple polymers in the same device. For this process, the polymers are chosen such that the refractive index between them is as high as possible. We simulate the SZ 2080 polymer with a refractive index of ≈1.5^3^ and the ma N 1400 Series polymer with a refractive index of 1.608.^4^

**Figure 7:**
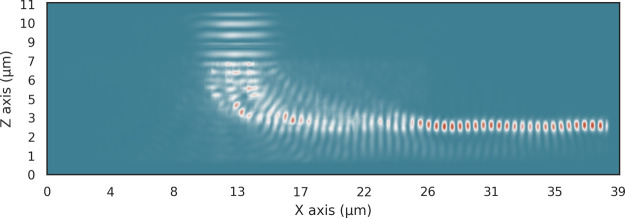
Energy distribution of the 3D two-polymer vertical coupler with a voxel height of 500 nm and a final attenuation of −9 dB.

While the results for the 2D case are similar to those of the single material coupler, as indicated in Figure 4, the 2.5D design achieves a coupling efficiency of −17 dB or around 2%. However, to increase the theoretical efficiency even further, we use the full 3D design space of the two polymer device. The results in Figure 7 demonstrate the effectiveness of this design space by coupling over 12% of the incoming light into the single-mode waveguide. Much of the lost energy is due to the relatively large feature size of 500 nm. However, we wanted to present a device that can, in theory, be fabricated with the current state of the art in 2PP printing.

## Conclusions

In this work, we present a memory efficient implementation of reverse-mode automatic differentiation, which enabled the integration of arbitrary constraints into the inverse design process of PICs. Our approach is based on the quantization of the design parameters and the application of the straight-through gradient estimator to backpropagate gradients through the FDTD simulation. We demonstrated the capabilities of our method by optimizing the design of several polymer-based vertical couplers. These devices are core components of PICs, and our results indicate the potential of multipolymer 2PP for their fabrication. Our method accelerates the development of polymer-based PICs due to its generality and usability.

## Supporting Information Available

Validation of the FDTD solver against established open-source software; runtime comparison between GPU and CPU implementations; details of 2PP fabrication constraints; optimization details including quantization mapping algorithms; and robustness analysis of coupling devices under various perturbations (PDF)

## Footnotes

1 The code is publicly available at https://github.com/ymahlau/fdtdx.

2 The simulation calculations in forward and backward direction also produce intermediate tensors, which require additional memory. Furthermore, the cumulative gradients need to be kept in memory.

3 The exact refractive index at 1550 nm of the resist depends on the degree of polymerization which itself depends on the exact printing parameters.^2^ Also see our brief section on 2PP printing in the Supporting Information.

4 The Cauchy equation for the ma N 1400 Series is .^41^
