# Supplementary material for: Quantized Inverse Design for Photonic Integrated Circuits
Source: ACS Omega. 2025 Jan 27;10(5):5080–6. doi: 10.1021/acsomega.4c10958 (PMC11822695; doi:10.1021/acsomega.4c10958)
Supplement: Supplementary file 1 — ao4c10958_si_001.pdf [file ao4c10958_si_001.pdf]

Quantized Inverse Design for Photonic Integrated Circuits – Supporting Information

Frederik Schubert,[*,†,¶] Yannik Mahlau,[*,†,¶] Konrad Bethmann,[†] Fabian Hartmann,[†] Reinhard Caspary,[‡] Marco Munderloh,[†] Jörn Ostermann,[†] and Bodo Rosenhahn[†]

†*Institute for Information Processing, Leibniz University, Hannover, Germany*

‡*PhoenixD, Leibniz University, Hannover, Germany*

¶*Equal Contribution*

E-mail: schubert@tnt.uni-hannover.de; mahlau@tnt.uni-hannover.de

Validation of the FDTD Solver

We validate our solver against the established open source FDTD software Meep[1] and use the random scatterer dataset by Augenstein et al.[2]. For this experiment, a randomly generated three-dimensional scattering object with refractive index 1.5 is placed inside a 6.12 µm $\times$ 6.12 µm $\times$ 6.12 µm volume. The simulation volume has a resolution of 40 nm with the outer 12 voxels being perfectly matched layers. At the bottom of the simulation volume, a planar wave with wavelength 1 µm is positioned. The source is active for the the first 10 periods of the simulation and then turned off. Afterwards, the simulation is run until all the fields are fully decayed, which takes about 100 fs. Figure S1 shows the central slices of the normalized steady state distribution of the E_z field. Our solver is able to predict the field distribution to a normalized L1 error of 2.1%.

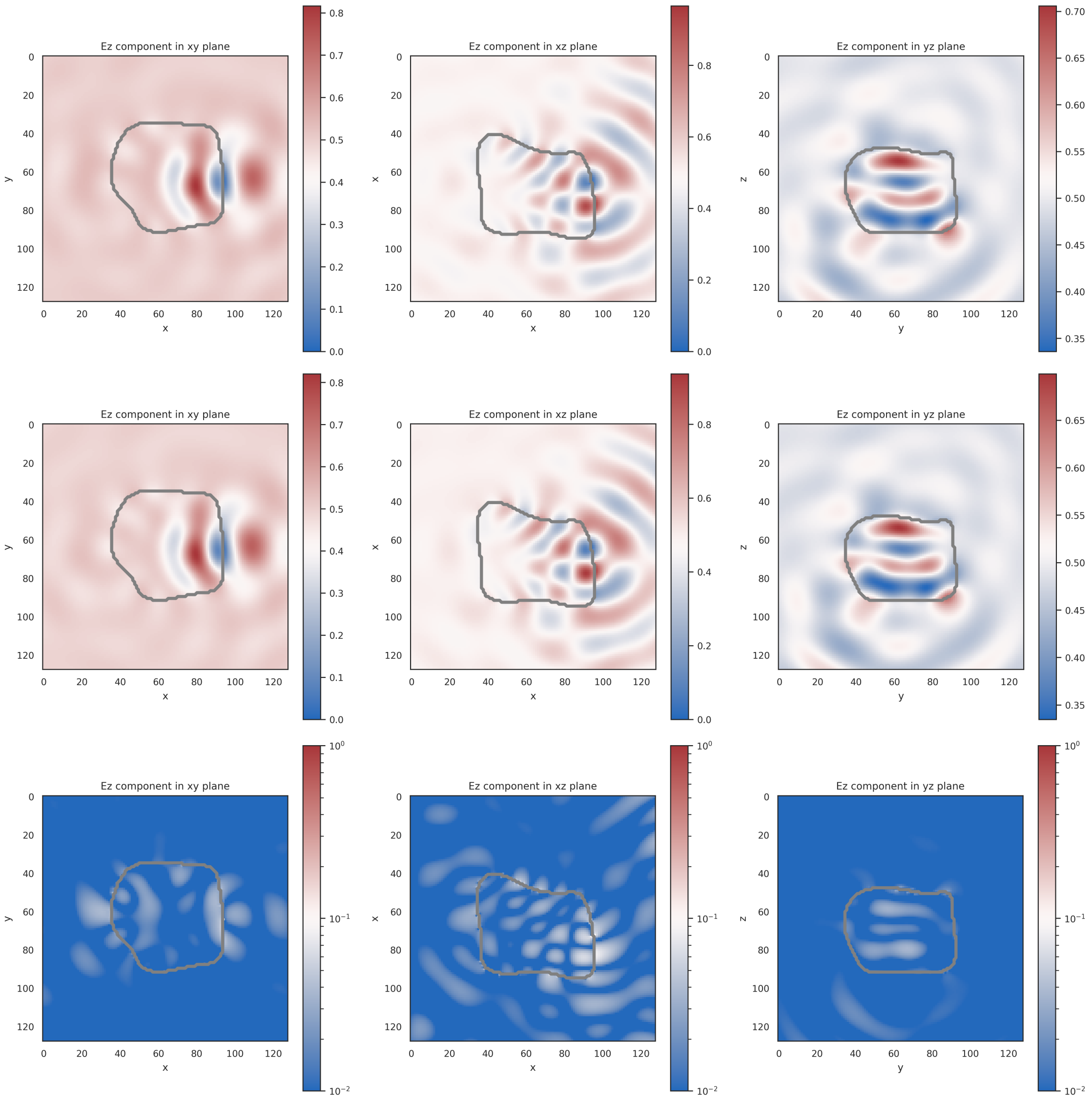

Figure S1: Detailed comparison between the predicted E_z field distributions (top) and the actual, or ground truth, E_z field distributions (middle), illustrating the accuracy of our FDTD solver in simulating electromagnetic wave propagation. The normalized absolute difference is shown in the bottom row.

To exemplify the speed gains enabled by running FDTD simulations on graphics cards, we compare the simulation runtime using a C++ implementation for CPU and GPU. The only previous previous implementation of automatic differentiation by time reversibility used plain C++.[3] In contrast, our new implementation is able to leverage the parallelization capabilities of modern graphics cards. For this demonstration, we performed the simulation of the random scatterer at different resolutions. Starting from 40 nm, we increased the

Table S1: Runtime comparison of the FDTD method on GPU and CPU using JAX and C++ for a simulation volume of 6.12 µm × 6.12 µm × 6.12 µm. All simulations were run for 200 fs.

**Resolution**	**Cells**	**Steps**	**CPU (C++)**	**GPU (JAX, ours)**
40 nm	3.5×10^6	2623	5 min 5 s	7.3 s
20 nm	28.6×10^6	5245	1 h 10 min	1 min 14 s
10 nm	229.2×10^6	10,490	18 h 49 min	16 min 41 s
5 nm	1833.7×10^6	20,980	97 h 18 min	3 h 19 min

resolution up to 5 nm resulting in 512 times larger simulations due to the cubic scaling in three-dimensional space. The results are displayed in Table S1. For the CPU simulation, we used the Meep framework[1] on a AMD EPYC 4137MHz hardware. Note that Meep itself could be configured to run faster using the MPI interface, but for this experiment we intend to use plain C++ code to give a comparison to the previous implementation of automatic differentiation by time reversibility. The GPU simulations were run on a single NVIDIA A100 for resolutions up to 10 nm and four NVIDIA A100 GPU for the resolution of 5 nm due to the larger memory requirements.

2PP Fabrication Constraints

Though the coupling example introduced by Shen et al.[4] demonstrates the potential of silicon-based PICs, the fabrication of such devices is time-consuming and expensive. As an alternative, we consider polymer-based PICs, which can be fabricated using two-photon polymerization (2PP).

Conventional laser lithography uses a photoresist which absorbs the laser radiation directly. The absorption of a photon triggers a photo initiator molecule, which starts the cross-linking chain reaction of monomers in the whole cross section volume of laser beam and photoresist, see Fig. S2 left. 2PP in contrast uses a femtosecond laser with long wavelength for which the photoresist is transparent. However, the ultrashort pulse of such a laser comes with extremely high peak power. The optical power level of the laser is carefully

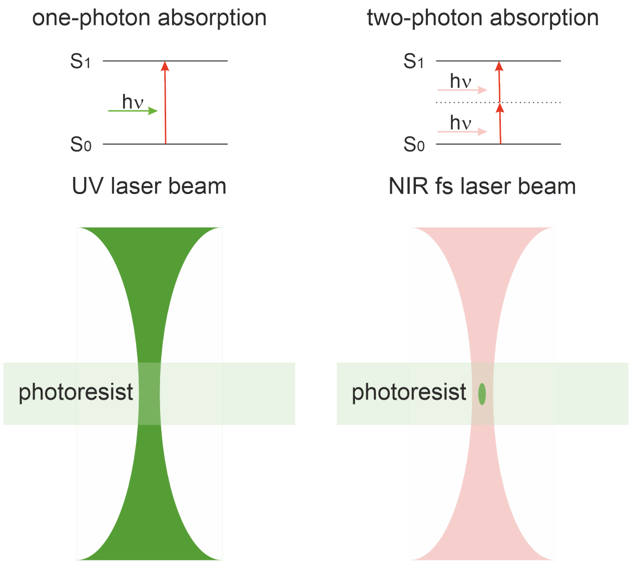

Figure S2: Comparison of conventional laser lithography (left) with 2PP printing (right).

adjusted so that in the very center of the laser focus the optical intensity exceeds the non-linear threshold for two-photon absorption which triggers the photo initiator molecules as illustrated in Fig. S2 right. Therefore, polymerization takes place only in a tiny volume in the center of the laser focus. This allows for a free voxel-based 3D printing process inside the volume of the photoresist without the need of support structures and at a resolution far beyond the diffraction limit of the optical system.[5] The non-linear process can actually involve the simultaneous absorption of multiple photons. It is thus sometimes called multi-photon polymerization instead of 2PP.

Resulting from this manufacturing technique, multiple constraints regarding the feasibility of designs arises. Firstly, feasible designs have to adhere to a minimum feature size. We enforce this constraints by optimizing a discrete voxel grid with cells of side length 500 µm. Post-2PP fabrication requires removal of unpolymerized photoresist through a washing process. Consequently, the 3D design must avoid fully enclosed cavities that would prevent complete photoresist removal. Lastly, another constraint is that all structural elements must maintain physical connectivity to the ground. This constraint arises due to the three-dimensional fabrication capabilities since two-dimensional designs are always guaranteed to be connected to the ground. The quantization mapping enforcing these new constraints is

Algorithm S1: 2.5D Quantization Mapping

Input: Latent parameters θ, valid permittivities $\mathcal{P}$

Output: Quantized parameters $\hat{\theta}$

```
// Filter for valid configurations
```

$\hat{\mathcal{P}} \leftarrow \emptyset$;

for $p \in \mathcal{P}^{|Z|}$ **do**

  if $\nexists i : (p_i = 1 \textit{ and } \exists j > i : p_j \neq 1)$ **then**

    $\hat{\mathcal{P}} \leftarrow \hat{\mathcal{P}} \cup \{p\}$;

```
// Find closest configuration
```

for $(x, y) \in X \times Y$ **do**

  for $p \in \hat{\mathcal{P}}$ **do**

```
    // Difference of voxels adjacent in z
```

    $d_p \leftarrow \sum_{z=1}^{|Z|-1} |(p_z - p_{z+1}) - (\theta_{x,y,z} - \theta_{x,y,z+1})|$;

```
    // Difference of average permittivity
```

    $d_p \leftarrow d_p + \frac{1}{|Z|} |\sum_{i=1}^{|Z|} \theta_{x,y,z} - \sum_{i=1}^{|Z|} p_z|$;

  $\hat{\theta}_{x,y} \leftarrow \arg\min_{p \in \mathcal{P}^{|Z|}} d_p$;

return $\hat{\theta}$;

discussed in the sections below.

Optimization Details

Building upon the 2D quantization approach outlined in **??**, we develop a 2.5D quantization mapping displayed in Alg. S1 that enforces a pillar structure. The process begins by determining all valid configurations, followed by calculating the distance between each pixel and possible configuration. Our distance metric does not only consider average permittivity, but also accounts for permittivity variations between voxels along the z-axis. This approach ensures that the quantization accurately captures the structural characteristics represented by the latent parameters.

In 3D designs, a simple quantization mapping might produce invalid structures where voxels may not be connected to the ground or air cavities within the design are present. To

Algorithm S2: 3D Quantization Mapping

Input: Latent parameters θ, valid permittivities $\mathcal{P}$
Output: Quantized parameters $\hat{\theta}$
for $(x, y, z) \in X \times Y \times Z$ **do**
    $\hat{\theta}_{x,y,z} \leftarrow \arg\min_{p \in \mathcal{P}} |\theta_{x,y,z} - p|$;
`// Remove enclosed cavities`
while $\exists \hat{\theta}_{x,y,z} = 1$ *not connected to outside* **do**
    for $(x, y, z) \in X \times Y \times Z$ **do**
        if $\hat{\theta}_{x,y,z} = 1$ *and not connected to outside* **then**
            $\hat{\theta}_{x,y,z} = p_i, \;\; p_i \in \mathcal{P}, p_i \neq 1$;
`// Eliminate structurally unsupported polymer voxels`
for $(x, y, z) \in X \times Y \times Z$ **do**
    if $\hat{\theta}_{x,y,z} \neq 1$ *and not connected to ground* **then**
        $\hat{\theta}_{x,y,z} = 1$;
return $\hat{\theta}$;

ensure feasibility, we implement a two-step process. Firstly, we fill any enclosed air cavities with material and afterwards remove all unsupported structures. This approach guarantees a valid design since the removal of unsupported structures cannot create new air cavities. The complete procedure is detailed in Alg. S2.

To demonstrate the robustness of our stochastic optimization we provide the full optimization curve for 5 seeds per design space in Fig. S3. Each iteration takes around 110 s on a NVIDIA A100 which results in a total wall-clock time of about 15 hours. The peak memory requirement of this optimization is about 14GB. Therefore, it would also be feasible to run this optimization on a consumer-grade graphics card like the NVIDIA RTX 4060 TI.

Using the fully optimized designs, we perform an extensive analysis on the influence of translation and rotational errors in the experimental setup, as well as the distribution under wavelength variations of the source. The results of this analysis are displayed in Fig. S4. All of the optimized structures are robust to minor changes in translation, rotation and wavelength. A rotation of 5 degrees, translation of 2 µm or a wavelength variation of 20 nm only result in a drop in efficiency of about -2 dB. Larger variations also lead to a more

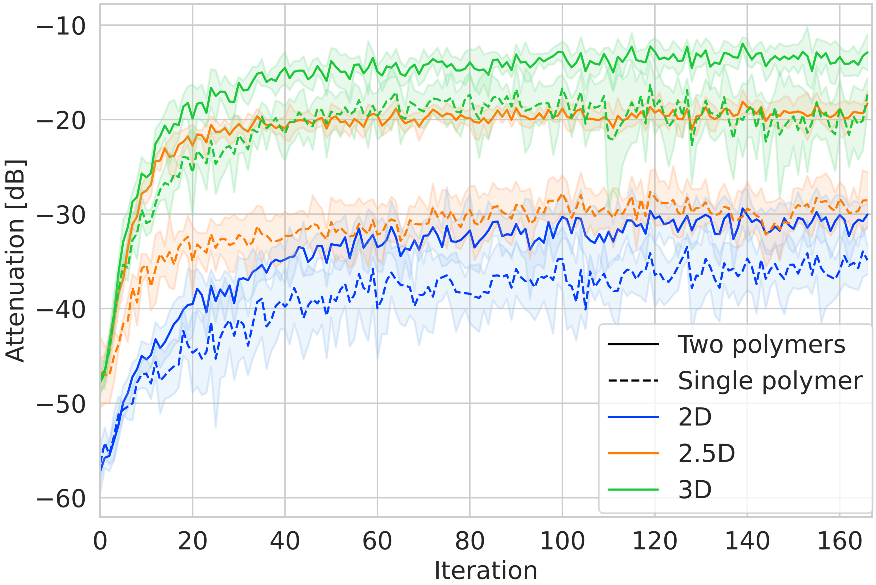

Figure S3: Average attenuation during the optimization of the coupling devices. The shaded area indicates the standard deviation over 5 random seeds and 3 random source calibration offsets per iteration.

drastic reduction in coupling efficiency. For example, a translation of 10 µm would place only about half of the source above the device, which results in an efficiency drop of 40 dB for the 3D multi-material devices. In conclusion, our devices are robust to small variations in the experimental setup, but larger variations should be avoided.

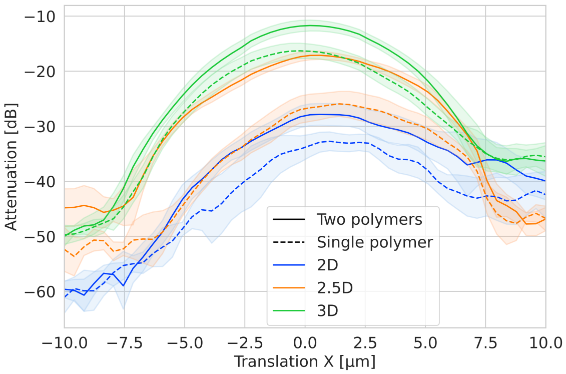

(a) Translation in X-axis

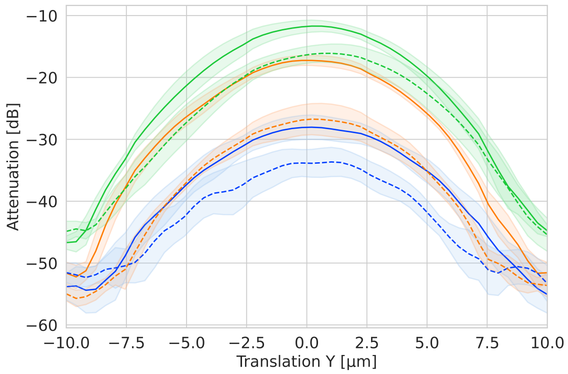

(b) Translation in Y-axis

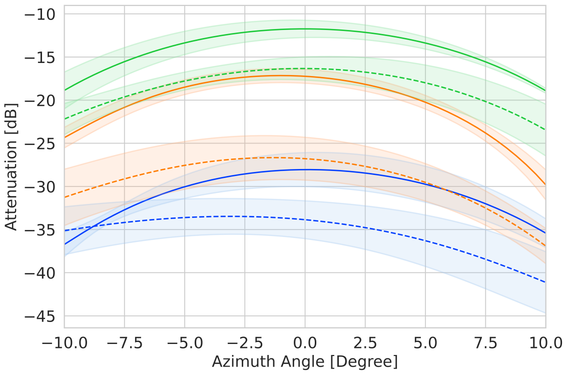

(c) Rotation of Azimuth angle

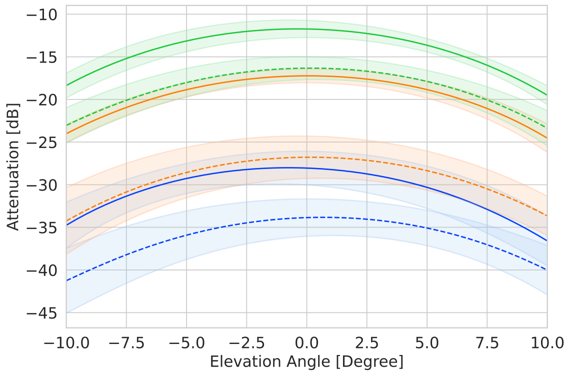

(d) Rotation of Elevation angle

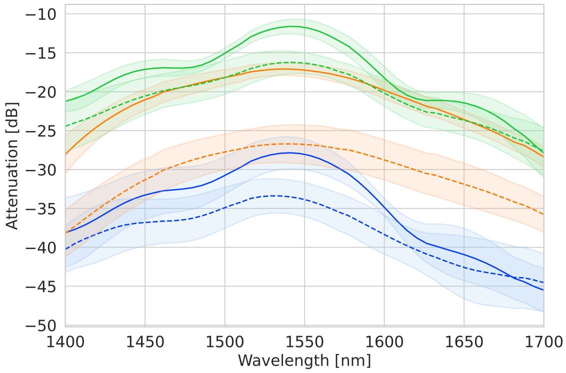

(e) Variation of Wavelength.

Figure S4: Analysis of the fully optimized coupling devices in 2D, 2.5D and 3D using either a single or two different polymers variations of the incoming light. The source is translated in X- and Y-axis and rotated using the azimuth and elevation angle. Additionally, the influence of the source wavelength on the coupling efficiency is evaluated. The mean and standard deviation are calculated over five random seeds.

References

(1) Oskooi, A. F.; Roundy, D.; Ibanescu, M.; Bermel, P.; Joannopoulos, J.; Johnson, S. G. Meep: A flexible free-software package for electromagnetic simulations by the FDTD

method. *Computer Physics Communications* **2010**, *181*, 687–702.

(2) Augenstein, Y.; Repän, T.; Rockstuhl, C. A Neural Operator-Based Surrogate Solver for Free-Form Electromagnetic Inverse Design. *ACS Photonics* **2023**, *10*, 1547–1557.

(3) Tang, R. J.; Lim, S. W. D.; Ossiander, M.; Yin, X.; Capasso, F. Time Reversal Differentiation of FDTD for Photonic Inverse Design. *ACS Photonics* **2023**, *10*, 4140–4150.

(4) Shen, B.; Wang, P.; Polson, R.; Menon, R. Integrated Metamaterials for Efficient and Compact Free-Space-to-Waveguide Coupling. *Optics Express* **2014**, *22*, 27175.

(5) Haske, W.; Chen, V. W.; Hales, J. M.; Dong, W.; Barlow, S.; Marder, S. R.; Perry, J. W. 65 Nm Feature Sizes Using Visible Wavelength 3-D Multiphoton Lithography. *Optics Express* **2007**, *15*, 3426.
